# Management of Denys-Drash syndrome: A case series based on an international survey 

**DOI:** 10.5414/CNCS109515

**Published:** 2018-11-12

**Authors:** Laurence Gariépy-Assal, Rodney D. Gilbert, Aleksas Žiaugra, Bethany Joy Foster

**Affiliations:** 1 Department of Pediatrics and Department of Epidemiology, Biostatistics and Occupational Health, Faculty of Medicine, McGill University, Montreal, QC, Canada,; 2 Department of Pediatric Nephrology, Faculty of Medicine, University of Southampton, Southampton, UK,; 3 Department of Internal Medicine, Faculty of Medicine, Health Sciences Centre, Postgraduate Medical Education, St. John’s, NL, and; 4 Department of Pediatric Nephrology, Faculty of Medicine, McGill University, Montreal, QC, Canada

**Keywords:** diffuse mesangial sclerosis, Wilms’ tumor, nephrectomy, management, genetic renal disease

## Abstract

Denys-Drash syndrome (DDS), a condition caused by mutations in the tumor-suppressor gene WT-1, is associated with a triad of disorders: ambiguous genitalia, nephrotic syndrome leading to end-stage renal disease (ESRD), and Wilms’ tumor. Given the variable disease course, management is challenging. We aimed to describe the evolution of DDS and the range of management strategies by summarizing the clinical courses of cases collected from a questionnaire sent to the international pediatric nephrology community. 15 respondents provided information on 23 patients; 21 DDS cases were confirmed and analyzed. At DDS diagnosis, 6 patients had a Wilms’ tumor (group A) and 15 had no Wilms’ tumor (group B). Three group A patients had unilateral nephrectomy. Two of these still had renal function, with no second tumor, at 36 months and 16 years of age, and 1 progressed to ESRD. Three had bilateral nephrectomy before ESRD. Eight group B patients progressed to ESRD, all of whom later had all renal tissue removed. Two group B patients subsequently developed a unilateral Wilms’ tumor and had bilateral nephrectomy pre-ESRD. Three had bilateral nephrectomy prior to reaching ESRD without ever having a Wilms’ tumor. Two group B patients remained tumor-free with renal function at last follow-up. Two main management approaches were taken: pre-emptive nephrectomy prior to ESRD and conservative surveillance. Based on the known risks associated with ESRD in infants and young children, the variable course of DDS, and the relatively good prognosis associated with Wilms’ tumor, a guiding principle of preservation of renal function is most logical. Most would advocate bilateral prophylactic nephrectomy after ESRD is reached due to the high tumor risk, which is likely heightened after transplant.

## Introduction 

Denys-Drash syndrome (DDS) is a rare genetic condition caused by mutations in WT-1 (chromosome band 11p13), a tumor-suppressor gene involved in gonadal development [1]. DDS is associated with a triad of disorders: ambiguous genitalia, nephrotic syndrome leading to end-stage renal disease (ESRD), and Wilms’ tumor [2]. All patients with DDS have renal histologic findings consistent with diffuse mesangial sclerosis (DMS) or, rarely, focal segmental glomerulosclerosis (FSGS) [3]. 

When making a management plan, the natural history of DDS must be considered. Patients with DDS typically progress to ESRD by the age of 4 years [4] and are at high risk of developing Wilms’ tumor [5]. However, the clinical course is highly variable, making the outcome of an individual patient difficult to predict. Therefore, management of patients with DDS is challenging; optimal management strategies have not been established. Two main management strategies have been proposed: bilateral nephrectomy only after progression of nephropathy to ESRD [6], or prophylactic bilateral nephrectomy prior to progression to irreversible renal failure to avoid Wilms’ tumor development and potentially shorten total duration of dialysis [7]. Surveillance for Wilms’ tumor using serial imaging studies is suggested in patients for whom a conservative approach is taken [3]. When a Wilms’ tumor is detected, some have performed immediate bilateral nephrectomy, whereas others advocate nephron-sparing surgery to preserve renal function as long as possible [8]. 

The approach that offers optimal outcomes remains undetermined. The objective of this study was to collect experiences of children with DDS from both our survey and prior published cases from the literature to inform a logical approach to management. 

## Methods 

We conducted an online survey (Survey Monkey) to collect information about DDS patients and their management from the international pediatric nephrology community between April and December 2016. Survey participants were solicited via the PedNeph Listserv. Participants were asked to provide information on presenting clinical features, genetic diagnosis, monitoring, surgical and medical management, age at each clinical event (presentation, ESRD, transplantation), and status (with native renal function, dialysis, transplantation, deceased) at last follow-up. Respondents could report details on one or multiple DDS patients. No identifying information was collected about patients or respondents. The name of the reporting center was collected to allow comparison of entries to avoid duplicates. DDS diagnosis was confirmed based on presence of a genetic mutation or, if genetic testing was not done, based on presence of at least 2 of 3 criteria (Wilms’ tumor, genital abnormality, evidence of renal disease based on DMS or FSGS on biopsy, presence of proteinuria, and/or progressive chronic kidney disease). Patients for whom DDS could not be confirmed were excluded from the analysis. Data were analyzed using Excel. Continuous variables were summarized with means and standard deviations, and categorical data were summarized with proportions to capture the different possible presentations and clinical pathways in DDS. The study was approved by the Research Ethics Board of the Montreal Children’s Hospital. Survey participants were informed in writing of the risks and benefits of participation; completion of the survey constituted consent. 

## Results 

Although 25 people accessed the survey, 10 did not actually complete the questionnaire. 15 respondents from the United States (n = 9; 9 patients), Canada (n = 2; 6 patients), the Netherlands (n = 1; 4 patients), Italy (n = 1; 2 patients), the United Kingdom (n = 1; 1 patient), and New Zealand (n = 1; 1 patient) provided information on 23 patients managed between 1995 and 2016. Two of the 23 were excluded because DDS could not be confirmed. Table 1 shows the clinical, genetic, and renal histopathological characteristics of each patient. 

Patients were divided into two groups based on presence (group A) or absence (group B) of Wilms’ tumor at DDS diagnosis. There were 6 patients with a Wilms’ tumor at DDS diagnosis, with a median age of 10 months (range 1 – 32 months). There were more than two times as many patients reported with no Wilms’ tumor at diagnosis. Of the 15 patients with no Wilms’ tumor at DDS diagnosis, age at diagnosis was reported for 13; these 13 patients ranged in age from 1 to 24 months at diagnosis, with a median age of 5 months. 

**Figure 1.**
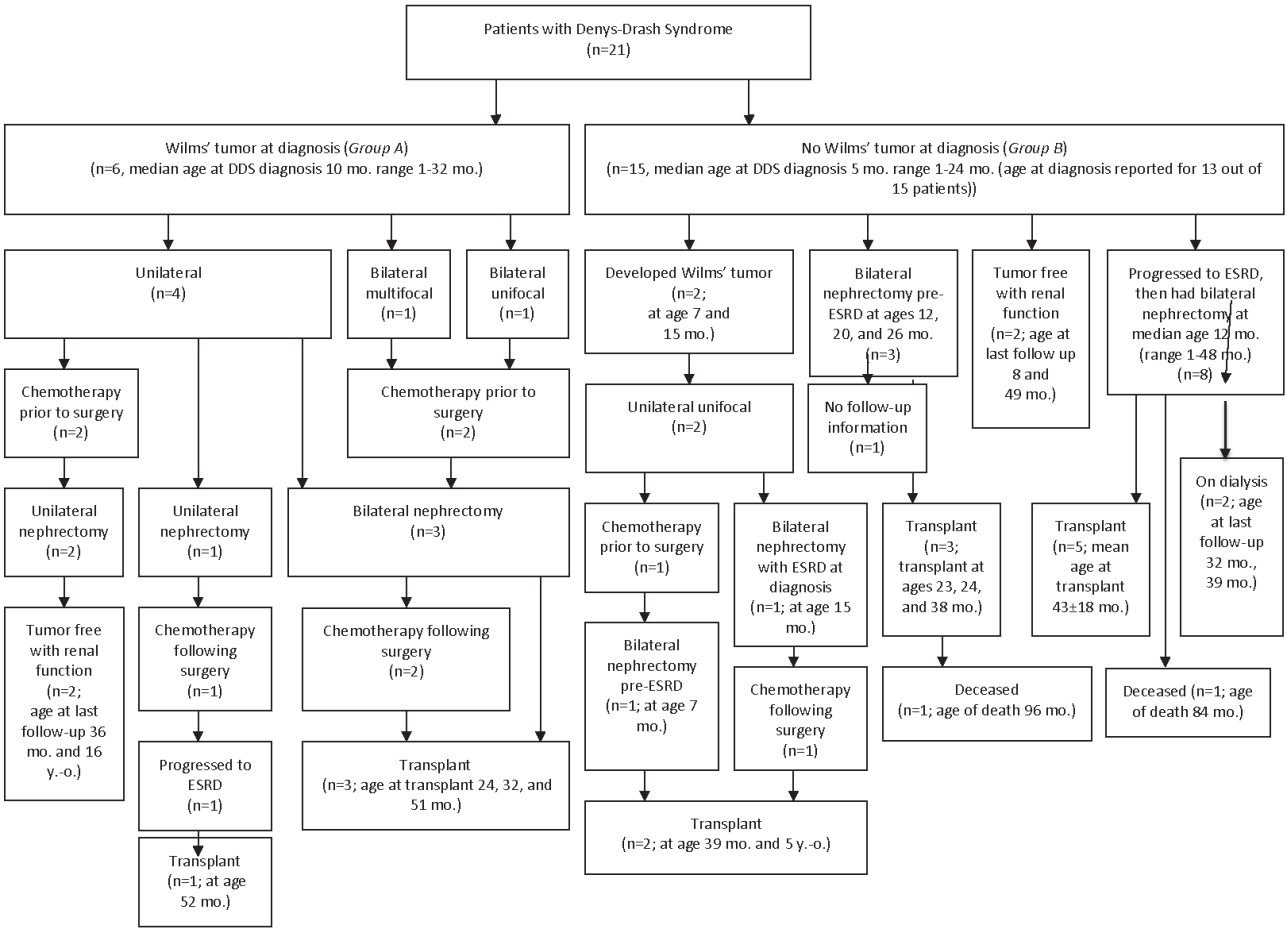
Flow chart illustrating the clinical pathways of all patients with a Wilms’ tumor at diagnosis (group A) and without a Wilms’ tumor at diagnosis (group B).

Figure 1 shows the clinical pathways of all patients, with their ages at various steps in progression of the disease, and how they were managed. All patients with Wilms’ tumor were managed with both chemotherapy and surgery. 

Four group A patients had a unilateral Wilms’ tumor at diagnosis. Three of those had unilateral nephrectomy; 2 of these still had renal function, with no second tumor, at 36 months and 16 years of age, and 1 progressed to ESRD (time to progression unknown). One of those with a unilateral Wilms’ tumor at DDS diagnosis at 32 months old had a bilateral nephrectomy (renal function at nephrectomy not available) and was treated with dialysis until transplant at 51 months of age. The 2 patients with bilateral Wilms’ tumor both underwent total bilateral nephrectomy as initial management. At nephrectomy, 1 of these patients had normal renal function (GFR > 90 mL/min), and the renal function of the other was not available. 

Among the 15 group B patients with no Wilms’ tumor at DDS diagnosis, 8 progressed to ESRD over a median of 4 months (median age at ESRD 7.5 months; IQR 3.5 – 29.0; range 1 – 40 months) and subsequently had all renal tissue removed (median age 12 months); 1 died post nephrectomy. Three group B patients had a bilateral nephrectomy pre-ESRD (ages 12, 20, and 26 months); 1 died post-transplant. Two group B patients developed a unilateral Wilms’ tumor, and both underwent bilateral nephrectomies; 1 had stage 4 chronic kidney disease, and the other had ESRD at the time of Wilms’ tumor detection at 7 and 15 months of age, respectively. Both were treated with dialysis for 32 and 45 months, respectively, before transplantation at 39 months and 5 years of age. Finally, 2 group B patients were tumor-free with normal renal function at age 8 and 49 months at last follow-up. 

Among patients who had bilateral nephrectomy before ESRD, regardless of Wilms’ tumor status, the median age at nephrectomy was 23 months, the median duration of dialysis was 10 months (range 1 – 35 months), and the median age at transplant was 31 months (range 23 – 51 months). In contrast, of those who had bilateral nephrectomy after ESRD, the median age at nephrectomy was 12 months (range 1 – 48 months), the median duration of dialysis was 27 months (range 7 – 78 months), and the median age at transplant 36 months (range 25 – 63 months). The 2 patients who developed a Wilms’ tumor during monitoring were transplanted 32 and 45 months after Wilms’ diagnosis. 

### Cases in the literature 

Past reports focused on the management of DDS with Wilms’ tumor at diagnosis or with advanced renal failure [7, 8, 9, 10, 11, 12]. A total of 39 cases were reported in six different publications. 15 of these cases had no Wilms’ tumor at diagnosis, of whom 11 had bilateral nephrectomy after reaching ESRD, 2 rapidly developed ESRD and died before surgery, and 2 had normal kidney function and were closely monitored with repeated abdominal ultrasounds. One of the two with normal kidney function remained tumor-free, with renal function at age 9 years, and the other died (no autopsy performed, cause of death not specified). 17 patients had a unilateral Wilms’ tumor at presentation; of these, 7 had a unilateral nephrectomy, 3 had ESRD and underwent bilateral nephrectomy, 1 underwent nephron-sparing surgery, 3 died in ESRD before surgery, and 3 had unilateral nephrectomy followed by prophylactic removal of the second kidney after 1 – 11 months pre-ESRD. Finally, 7 patients had bilateral Wilms’ tumor at diagnosis. Of these, 1 died before surgery, 2 had ESRD and underwent bilateral nephrectomy, and 4 underwent nephron-sparing surgery. Of those who had nephron-sparing surgery, 3 eventually had total nephrectomy after progression to ESRD (age at total nephrectomy was not specified). 

## Discussion 

This report summarizes the varied clinical trajectories and different strategies used in the in the management of patients with DDS. Two main approaches were taken: pre-emptive nephrectomy and conservative surveillance. 

### Pre-emptive nephrectomy 

Four of the 21 patients captured by our survey had bilateral nephrectomy prior to ESRD – 3 never having had a Wilms’ tumor, and 1 with a unilateral Wilms’ tumor. The main arguments in favor of prophylactic bilateral nephrectomy are that this approach avoids the potential complications of Wilms’ tumor, and may reduce the total duration of dialysis before a transplant can be done. 

Because most would recommend an interval of at least 1 year following completion of treatment for Wilms’ tumor before kidney transplantation [13], it is possible that a child could require a year of dialysis before transplant. By comparison, if a bilateral nephrectomy were done before a Wilms’ tumor presented, the child could be exposed to dialysis for as little as 6 – 8 weeks between nephrectomy and transplantation. We observed a shorter median time on dialysis for patients who had bilateral nephrectomy before ESRD (10 months; range 1 – 35 months) compared to those who had bilateral nephrectomy after ESRD (27 months; range 7 – 78 months) – although this difference was not statistically significant, and other confounding factors may have contributed. Dialysis is associated with substantially higher mortality rates, and worse quality of life compared with conservative management of chronic kidney disease or kidney transplant [14, 15]. Minimizing the duration of dialysis is desirable. Prophylactic nephrectomy may also provide the advantage of initiating dialysis in a controlled setting in which a functional peritoneal dialysis catheter, with a completely-healed insertion site, is in place. Moreover, a prophylactic nephrectomy prior to onset of severe nephrotic syndrome may help avoid the complications associated with nephrotic syndrome, including thromboembolic events and poor wound healing [16]. 

However, although pre-emptive bilateral nephrectomy may offer shorter time on dialysis and avoidance of complications of nephrotic syndrome, this must be weighed against the increased risks associated with dialysis initiated at a young age. The risks associated with dialysis, and with transplantation, are amplified in younger children, with the highest mortality risks in the youngest children [17]. In addition, the first 2 – 3 years of life are a critical period for growth and cognitive development [18]; ESRD in infancy and early childhood may have an irreversible negative impact on growth and development [19]. Furthermore, most centers require that children reach 10 kg before becoming eligible for transplant; bilateral nephrectomy prior to 10 kg will likely prolong dialysis, rather than reducing its duration. 

### Conservative management 

In both our survey and the literature, conservative management was the most common approach. One of the principal arguments in favor of a conservative approach is that the risks associated with dialysis and transplantation decrease with increasing age. Dialysis and transplantation are less technically difficult in older children with larger body size. In addition, dialysis-associated infection rates are lower in older than in younger children, as are mortality rates [17, 20]. Among patients recorded in the United States Renal Data System (2005 – 2010), the mortality rate for children < 5 years of age at dialysis initiation was 83.4 per 1,000 person-years of dialysis compared with 25.9 per 1,000 person-years of dialysis for patients 5 – 20 years at dialysis initiation [21]. Infants have the highest mortality rates of all children. In the North American Pediatric Renal Trials and Collaborative Studies (NAPRTCS) registry, between 1992 and 2010, infants initiating dialysis at < 2 years of age had a 3-year survival of 75.1%, compared with 89.6% for children 2 – 5 years old at initiation [22]. The 2016 United States Renal Data System (USRDS) annual report estimated 5-year survival for children with ESRD at 84% for those aged 0 – 4, 93% for those aged 5 – 9, and 96% for those aged 10 – 13. Age disparities in 1-year mortality rates are even larger, at 63 per 1,000 patient-years for children aged 0 – 4 and 26 per 1,000 patient-years for those aged 5 – 9 [23]. 

Younger age at transplantation is also associated with poorer outcomes than at older age. Among living-donor transplant recipients recorded in the NAPRTCS registry (1996 – 2010), 3-year survival for infants < 2 years old was 95.9% compared with 97.4% for children 2 – 5 years old. For recipients of deceased-donor grafts, the difference was greater, with a 3-year survival of 93.4% for infants < 2 years old compared with 96.3% for children 2 – 5 years old, and 98.9% for those 6 – 12 years old [24]. 

The developmental impact of ESRD in infancy must also be considered. A significant portion of linear growth [25] and ~ 50% of postnatal brain growth [26] occurs during the first year of life. Growth failure is common in ESRD, particularly among infants [27]. ESRD also appears to affect neurocognitive development, with the largest deficits in children who were youngest at ESRD onset [19]. Prophylactic bilateral nephrectomy at a very young age may increase the risk of growth restriction and impaired neurodevelopment. 

One of the primary reasons that prophylactic bilateral nephrectomy is recommended may be the desire to avoid cancer. However, it is worth noting that the prognosis for Wilms’ tumor is generally excellent. Even between 1974 and 1978, the 2-year survival rate for early stage Wilms’ tumor treated with 6 – 15 months of chemotherapy (actinomycin D and vincristine) was 95% [28]. More recently (1990 – 2010), the 5-year survival of non-metastatic Wilms’ tumor was estimated at 93.8% [29]. Children with DDS who are screened regularly are likely to have Wilms’ tumor identified early, and therefore have a better prognosis; stage I favorable histology Wilms’ tumor has a 16-year survival rate of 97.6% [30]. 

Perhaps the most compelling reason to favor conservative management is the variable course. DDS patients who had not developed Wilms’ tumor and maintained renal function well into childhood have been reported [31], including a child who remained tumor-free with normal kidney function to at least age 9 years [11]. 

### Suggested approach to DDS management 

The management of DDS should be individualized and will always involve careful balancing of risks. However, based on the known risks associated with ESRD in infants and young children, the variable course of DDS, and the relatively-good prognosis associated with Wilms’ tumor, a guiding principle of preservation of renal function is most logical. Once ESRD is reached, most agree that bilateral nephrectomy should be performed to eliminate the risk of a tumor in the future. This was the most common approach reported in our survey. Many factors must be considered in planning DDS management, including age of the child, amenability of the tumor to a nephron-sparing approach, complications associated with the nephrotic syndrome that may occur with DDS, control of hypertension, rate of progression of renal failure, and Wilms’ tumor histology. The Wilms’ tumor recurrence rate is 15% for favorable histology and 50% for anaplastic Wilms’ tumor [32]. Regular surveillance for Wilms’ tumor is necessary in these children, although the optimal frequency is unknown. 

### Limitations 

There are a number of limitations to our study. A case series has no comparator group and therefore can only describe the clinical presentation and course of cases as they were managed, rather than compare outcomes with different management approaches. In addition, the number of participants and the number of reported cases was small. The small sample reflects, in part, the rarity of DDS. Those who chose to report may have been biased towards a particular management approach and/or may have selected a biased sample of cases to report. It is possible that the cases reported here and the clinical pathways described do not reflect the full spectrum of DDS. Furthermore, given small numbers, it is not possible to draw firm conclusions about optimal management based on our survey data. The proposed recommendations are based primarily on known risks associated with ESRD [18, 19] and Wilms’ tumor [28, 30, 32]. Despite an uncertain course, overall prognosis is likely superior when renal function is preserved as long as possible, even if Wilms’ tumor develops. Finally, as in any retrospective study, the validity of the findings depends on the accuracy of the reported data – which are not verifiable [33]. Despite these limitations, this case series provides additional information on the clinical course and management strategies for patients with DDS. 

## Conclusion 

This study represents one of the largest collected series of DDS patients and provides updated information on management strategies used around the world. We also suggest an approach to management based on balance of risks. 

## Acknowledgment 

The authors are grateful to all members of the PedNeph Listserv who participated in the survey and shared information about DDS patients and their management. 

## Funding 

There was no funding to support this work. 

## Conflict of interest 

The authors have no conflict of interest to declare. 

**Table 1.** Characteristics and clinical background information of all patients included in the report.

Age at diagnosis (months)	Age at Wilms’ tumor detection (months)	Phenotypic sex	Karyotype	Genetic mutation	Biopsy finding	Genital abnormality	Hypertension	Protein/creatinine ratio at diagnosis (g/g)	Creatinine at diagnosis (mmol/L)	Renal function at nephrectomy
1	1	Male	N/A	N/A	N/A	Hypospadia proximal	No	2	N/A	N/A
7	7	Female	46, XX	Found, not specified	N/A	None	Yes	N/A	N/A	N/A
7	7	Male	46, XY	Found, not specified	DMS	Hypospadia proximal	Yes	10	54	Stage 1
12	12	Female	N/A	Found, not specified	N/A	None	Yes	14	18	Stage 1
14	14	Male	46, XY	Found, not specified	N/A	Hypospadia proximal, bilateral completely undescended testes (non-palpable), ambiguous genitalia	No	N/A	N/A	N/A
32	32	Male	46, XY	Exon 9 mutation	DMS	Bilateral completely undescended testes (non-palpable)	Yes	N/A	38	N/A
0	No WT	Male	46, XY	Exon 9 mutation	N/A	Hypospadia proximal, bilateral palpable inguinal testes	Yes	N/A	346	Stage 5
1	15	Male	46, XY	Found, not specified	DMS	Ambiguous genitalia	No	5	88	Stage 5
1	No WT	Male	46, XY	Found, not specified	N/A	Bilateral completely undescended testes (non-palpable)	Yes	1.0	177	Stage 5
1	No WT	Male	46, XY	Exon 8 mutation	N/A	Hypospadia proximal, bilateral completely undescended testes (non-palpable)	No	31.3	72	N/A
3	No WT	Male	46, XY	Found, not specified	DMS	Hypospadia proximal, unilateral completely undescended testis (non-palpable), ambiguous genitalia	No	4	60	Stage 3
4	7	Female	46, XY	Found, not specified	DMS	Normal female external genitalia	Yes	N/A	57	Stage 3
5	No WT	Male	46, XY	Exon 10 mutation	Nephrogenic rest	**A**mbiguous genitalia	Yes	N/A	28	Stage 3
6	No WT	Female	46, XX	Exon 9 mutation	DMS	Ambiguous genitalia	Yes	3.6	400	Stage 5
6	No WT	Male	46, XY	Exon 8 mutation	N/A	Hypospadia proximal, bilateral completely undescended testes (non-palpable), ambiguous genitalia	Yes	10	N/A	Stage 5
10	No WT	Male	46, XY	Found, not specified	DMS	Ambiguous genitalia	No	N/A	16	Stage 1
14	No WT	Male	N/A	N/A	FSGS	Bilateral completely undescended testes (non-palpable)	Yes	N/A	N/A	Stage 5
15	No WT	Male	46, XY	N/A	DMS	Bilateral completely undescended testes (non-palpable)	Yes	14,6	22	N/A
24	No WT	Male	46, XY	Intron 9 mutation	DMS	Hypospadia proximal, bilateral completely undescended testes (non-palpable), ambiguous genitalia	Yes	4	88	Stage 5
N/A	No WT	Male	46, XY	Exon 8 mutation	DMS	Hypospadia proximal, bilateral completely undescended testes (non-palpable)	No	N/A	N/A	Stage 5
N/A	No WT	Male	46, XY	N/A	DMS	Bilateral completely undescended testes (non-palpable)	Yes	N/A	442	Stage 5

N/A = not applicable; No WT = no Wilms’ tumor; DMS = diffuse mesangial sclerosis; FSGS = focal segmental glomerulosclerosis.
